# Absence of Low Internal Echoes as an Indicator of Non-aggressive Components in Hepatocellular Carcinomas: A Case Report

**DOI:** 10.7759/cureus.86616

**Published:** 2025-06-23

**Authors:** Ko Matsuura, Shoji Oura, Hitomi Matsuki, Yurie Kitano, Hiroshi Shintani

**Affiliations:** 1 Department of Gastroenterology, Kobe Tokushukai Hospital, Kobe, JPN; 2 Department of Surgery, Kishiwada Tokushukai Hospital, Kishiwada, JPN

**Keywords:** aggressive biology, differentiation degree, hepatocellular carcinoma, high internal echoes, low internal echoes

## Abstract

The differentiation degree of hepatocellular carcinomas (HCCs) well correlates with their aggressiveness. However, except for tumor size and mass border clarity, no preoperative images have been clarified to easily and accurately predict the differentiation degree of HCCs.A 74-year-old man with a liver mass was referred to our hospital. Computed tomography (CT) showed a round and well-circumscribed mass with a slightly lower Hounsfield Unit value than that of the surrounding liver parenchyma, and showed weak early enhancement and a washout pattern. Ultrasound showed an oval mass with very high internal echoes and enhanced posterior echoes. Magnetic resonance imaging of the mass showed slightly low signals on T1-weighted images, high intensity with focal low signals on fat-suppressed T2-weighted images, and weak early enhancement on subtraction images. Due to the elevated serum Protein Induced by Vitamin K Absence or Antagonist-II (PIVKA-II) level in addition to these image findings, the patient underwent a laparoscopic partial hepatectomy under the tentative diagnosis of HCC. Postoperative pathological study showed an oval mass, 65 mm in size, mainly composed of moderately differentiated HCC cells growing in a cord-like fashion with numerous pseudoglandular structures, a thin fibrous capsule encompassing the tumor, and focal areas of well-differentiated HCC cells growing in a thin-trabecular fashion with numerous lipid droplets. Clinicians should note that the absence of low internal echoes indicates the absence of aggressive components even in large HCCs.

## Introduction

Hepatocellular carcinomas (HCCs) are malignant diseases that often develop secondary to chronic liver diseases such as hepatitis B/C and cirrhosis. Although HCCs do not have a very high morbidity rate, they have a very high mortality rate, and the median survival of 6-20 months after diagnosis [1]. In addition to the highly aggressive nature of HCCs, their poor prognosis of HCCs is also attributable to their late detection. In short, small HCCs rarely cause specific symptoms, including para-neoplastic syndromes, and often lead to delayed diagnosis. It is, therefore, very important for physicians to identify patients at high risk for developing HCC and to promptly diagnose HCCs.

HCCs have a number of staging and prognostic scoring systems, such as the tumor, node, metastasis staging, the Okuda system [2], the Cancer of the Liver Italian Program score [1], and the Barcelona staging classification [3]. However, none of them, unfortunately, has become the global standard that surpasses the others. In addition, it is well known that various other factors can affect the survival of HCC patients, such as geographical factors [4], serum alpha-fetoprotein (AFP) level [5], variant estrogen receptors [6], and diabetes mellitus [7]. These facts strongly suggest that oncologists must appropriately evaluate each prognostic factor when deciding the treatment strategies and predicting the prognosis.

Like other solid malignancies, tumor histology, including histological differentiation degrees of HCCs, also has an impact on the survival of HCC patients [8]. Therefore, if the differentiation degree of HCCs can be predicted by routine imaging, it would also be beneficial for oncologists to decide treatment strategies. Hepatocytes receive blood flow from both the portal vein and the hepatic artery. The portal vein provides three times as much blood flow to the hepatocytes as the hepatic artery does. Therefore, the predominant blood supply to liver tumors is the portal vein in their early stages and changes to the hepatic artery as the tumors grow. It is, therefore, well known that contrast enhancement on images is generally slower in well-differentiated HCCs than in poorly differentiated HCCs due to the portal vein dominant blood supply to the well-differentiated HCCs [9]. In addition, it is also known that small, well-differentiated HCCs often have unclear borders. However, other than these image findings, the correlation between the degree of differentiation of HCCs and imaging findings is not well understood.

We herein report a large HCC case that had very high internal echoes and no poorly differentiated HCC components.

## Case presentation

**Figure 1:**
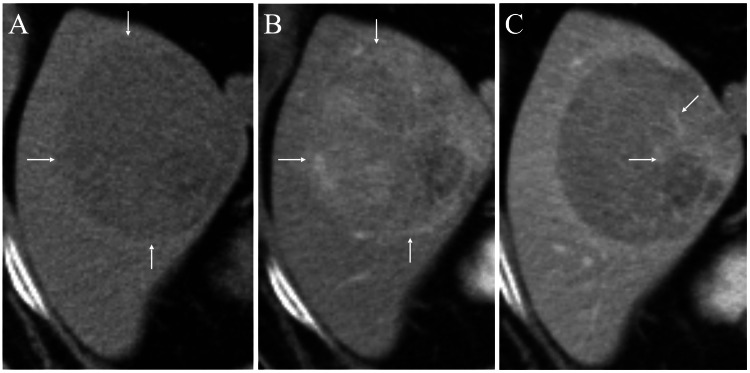
Computed tomography (CT) findings (A) Plain CT showed an oval mass (arrows), which had clear margins and a slightly lower Hounsfield Unit value than that of the surrounding liver parenchyma. (B) Enhanced CT showed a weak and heterogeneous early enhancement of the mass (arrows). (C) Enhanced CT showed a heterogeneous wash-out pattern of the mass and partially retained enhancement (arrows).

A 74-year-old man with a body mass index (BMI) of 26.1 had undergone transurethral resection (TUR) of early urothelial carcinoma of the bladder three times and was incidentally found to have a liver mass on computed tomography (CT) for a postoperative recurrence check of the bladder cancer 50 months after the last TUR. Although the patient had no chronic liver diseases such as hepatitis or cirrhosis, he had a history of long-term heavy drinking. CT showed a round and well-circumscribed mass, which had a slightly lower Hounsfield Unit (HU) value than that of the surrounding liver parenchyma (Figure 1A), weak early enhancement, and a washout pattern (Figures 1B-1C).

**Figure 2:**
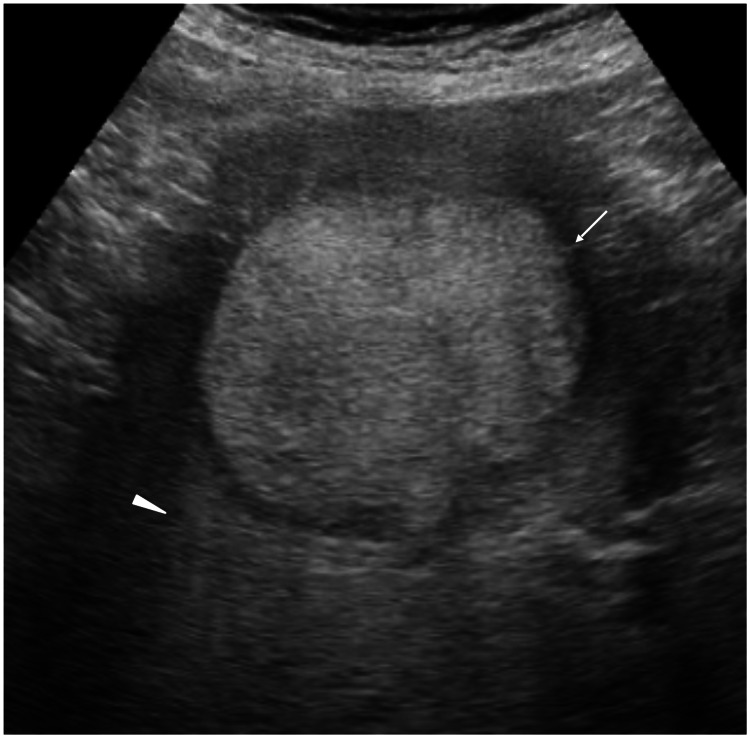
Ultrasound findings Ultrasound showed an oval mass with very high internal echoes, enhanced posterior echoes (arrowhead), and a presumed fibrous capsule (arrow).

The ultrasound (US) showed an oval mass with a surrounding presumed fibrous capsule, a very hyper-echoic pattern, and posterior enhancement, highly suggesting the mass to be HCC (Figure 2).

**Figure 3:**
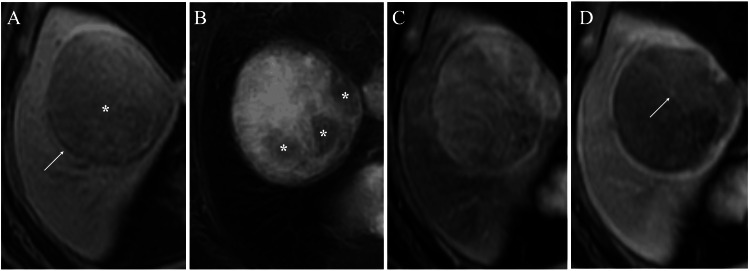
Magnetic resonance imaging (MRI) findings (A) The T1-weighted image showed an oval mass (asterisk) and the surrounding hypointense ring (arrow). (B) The fat-suppressed T2-weighted image showed that the mass had a mixed hypointense (asterisks) and weak hyperintense areas. (C) Subtraction image at 39 seconds after the contrast medium injection showed a weak early enhancement of the mass. (D) Subtraction image at 120 seconds after the contrast medium injection showed a wash-out pattern with residual slight enhancement of the mass (arrow).

MRI of the well-circumscribed mass showed slightly low signal intensity on T1-weighted images (Figure 3A), high signal intensity with focal low signals on fat-suppressed T2-weighted images (Figure 3B), and maximum but weak enhancement at 39 seconds (Figure 3C), with retained slight enhancement even at 120 seconds (Figure 3D) on subtraction images.

**Figure 4:**
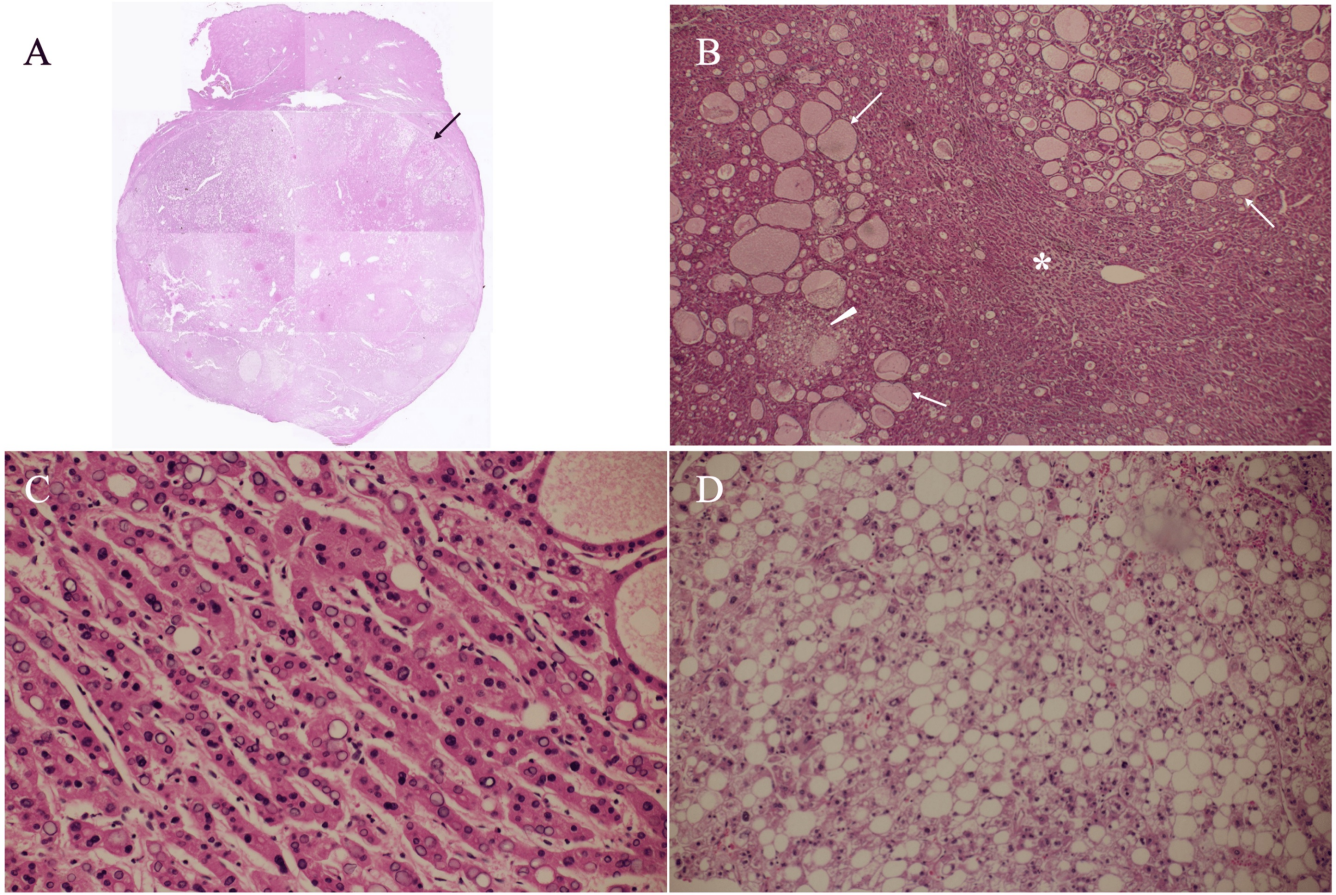
Pathological findings (A) Low-magnification view showed an oval mass surrounded by a thin fibrous capsule (arrow). (B) Slightly higher magnified view showed numerous pseudograndular structures (arrows), well-differentiated hepatocellular carcinoma components (asterisk), and a cluster of lipid droplets (arrowhead). (C) Magnified view showed well-differentiated hepatocellular carcinoma cells growing in a thin-trabecular fashion. (D) Magnified view showed numerous lipid droplets.

AFP and lectin-reactive fraction of AFP showed normal levels of 2.1 ng/mL and <0.5%, respectively. Protein Induced by Vitamin K Absence or Antagonist-II (PIVKA-II), however, showed a high level of 1426 ng/mL (reference range: 0-28.4 ng/mL). These images and laboratory findings led us to the preoperative diagnosis of HCC. The patient, therefore, underwent a laparoscopic partial hepatectomy. Postoperative pathological study showed an oval mass, 65 mm in size, mainly composed of moderately differentiated HCC cells growing in a cord-like fashion with numerous pseudoglandular structures, a thin fibrous capsule encompassing the tumor, and focal areas of well-differentiated HCC cells growing in a thin-trabecular pattern with numerous lipid droplets (Figure 4).

The patient was discharged on the 12th day after the operation and has been well for 24 months without any recurrences.

## Discussion

Backscattering of US waves determines internal echoes of masses. When cells or materials with completely or very similar acoustic impedance form a mass, there is little or no backscattering of US waves, resulting in anechoic or very low internal echoes, respectively. Conversely, differences in acoustic impedance of mass components generate backscattering of US, leading to high internal echoes. Therefore, homogeneity of acoustic impedance usually indicates high tumor proliferation potential, whereas heterogeneity indicates the opposite. In other words, tumors with low internal echoes are more aggressive than those with high internal echoes [10]. In short, regardless of tumor types, high internal echoes on US indicate favorable biology, while low internal echoes strongly suggest aggressive biology.

Fat has the lowest acoustic impedance in human bodies [11,12]. The presence of fat components in masses, therefore, always generates backscattering of US waves, leading to extremely high internal echoes [11]. In the early stages of HCC development, lipid droplets seen in fatty liver are often still present, mixed with well-differentiated HCC cells. However, in poorly differentiated HCCs, the aggressive cancer cells proliferate in an expansive manner and eliminate lipid droplets from the tumors. This patient was a long-term heavy drinker and consequently had fatty liver. Therefore, very strong high internal echoes were speculated to be caused by the extensive distribution of retained lipid droplets throughout the HCC [11,12] in this case, and highly suggested a favorable biology of this HCC.

It is well known that papillary and tubular lesions also make high internal echoes. The presence of micro-voids observed in papillary and tubular structures, including glandular and pseudo-glandular structures, also generates back scattering of US waves, leading to high internal echoes [13]. However, differences in acoustic impedance between cancer cells and micro-voids are generally smaller than those between cancer cells and fat components. It is, therefore, reasonable to conclude that the high internal echoes caused by pseudoglandular structures enhanced the very high internal echoes generated by the abundant fat cells present within the tumor in this case.

HCCs have four principal histological growth patterns: trabecular, solid, pseudoglandular, and macrotrabecular. Of these four growth patterns, the macrotrabecular pattern is associated with a worse prognosis [14]. The macrotrabecular growth pattern highly implies the presence of poorly differentiated HCCs. Like breast cancer with very low internal echoes [10], poorly differentiated HCCs generally have low internal echoes due to the less backscattering caused by HCC cells, consisting of relatively uniform cells with similar acoustic impedance, growing in an expansive pattern. HCCs often have a mixture of the four typical growth patterns and show low internal echoes at the aggressive components. We can, therefore, conclude that the absence of low internal echoes suggests the less aggressive nature of HCCs. Although this study is only a report of one case, considering the pathological findings of HCCs and the mechanism of internal echo formation by US, this finding should be applicable to all HCCs.

Since standard preoperative chemotherapy has not yet been established [15,16], prediction of HCC differentiation degree does not affect the decision whether to apply primary chemotherapy or initial local therapy to the HCC patients. However, prediction of the HCC differentiation degree may influence the selection of local therapy. When a good prognosis is highly expected before local therapy, there is an increased chance that surgery, i.e., the best therapeutic option for local control, will be selected [3].

## Conclusions

Many diagnostic physicians understand that well-differentiated HCCs often have lipid droplets in HCCs, leading to high internal echo formation. It is, however, extremely rare for physicians to preoperatively evaluate what pathological components correspond to the low internal echo parts within the tumor. Clinicians should note that the absence of low internal echoes indicates the absence of aggressive components even in large HCCs.
